# Halophilic *Aspergillus penicillioides* from athalassohaline, thalassohaline, and polyhaline environments

**DOI:** 10.3389/fmicb.2014.00412

**Published:** 2014-08-05

**Authors:** Sarita W. Nazareth, Valerie Gonsalves

**Affiliations:** Department of Microbiology, Goa UniversityTaleigao Plateau, India

**Keywords:** *Aspergillus penicillioides*, obligate, halophile, hypersaline, polyhaline

## Abstract

*Aspergillus penicillioides* is a true halophile, present in diverse econiches – from the hypersaline athalassohaline, and thalassohaline environments, to polyhaline systems, and in different geographical locations. Twenty seven isolates from these environments, were seen to be moderate halophiles, euryhaline in nature. They had an obligate need of a low a_w_ and were unable to grow on a regular defined medium such as Czapek Dox Agar, as well as on varied nutrient rich agar media such as Malt Extract, Potato Dextrose and Sabouraud Agar; however, growth was obtained on all these media when amended with 10% solar salt. In absence of added salt, the conidia either did not germinate, or when germinated, distortions and lysis were seen in the short mycelial forms; on media with salt, the mycelia and vesicles appeared normal.

## INTRODUCTION

*Aspergillus penicillioides* growth is favoured by low a_w_, and can grow even at an a_w_ of 0.68, which is in hibitory to most fungi (Tamura et al., 1999; Pitt and Hocking, 2009). *A. penicillioides* has been found in diverse habitats of low a_w_, such as the Dead Sea, solar salterns, mangroves, estuary (Wasser et al., 2003; Butinar et al., 2011; Gonsalves et al., 2012; Nayak et al., 2012; Nazareth et al., 2012), on foods such as grains, dried fruit, baked goods, salted fish and spices, as well as on binocular lenses and human skin (Andrews and Pitt, 1987; Tamura et al., 1999; Pitt and Hocking, 2009).

Organisms able to grow under conditions of low a_w_ and requiring NaCl, are known as halophiles (Kushner, 1978; Grant, 2004), distinguishing them from those merely able to grow at a low a_w_ caused by low moisture content, such as xerophiles (Andrews and Pitt, 1987; Tamura et al., 1999; Grant, 2004; Pitt and Hocking, 2009) or high osmotic pressures of sugar solutions, such as osmophiles (Tucker and Featherstone, 2011).

This paper reports *A. penicillioides* as a true halophile, present in diverse econiches of athalassohaline, and thalassohaline hypersaline environments, as well as polyhaline systems.

## MATERIALS AND METHODS

### ISOLATES

Twenty seven strains of *A. penicillioides* were tested, which were previously isolated from the Dead Sea water (DSw) and sediment (DSs) samples (Nazareth et al., 2012), from the estuary of Mandovi, Goa, on the West Coast of the Indian peninsula, surface and bottom waters (EMw_s_ and EMw_b_) and from sediment (EMs) samples (Gonsalves et al., 2012), from water samples from mangroves of Ribander, Goa (MRw) and from solar salterns at Santa Cruz (SCw), Goa, India (Nayak et al., 2012). The isolate numbers along with the sites of isolation are shown in **Table 1**.

**Table 1:** Isolates obtained from various econiches of different salinity.

Sampling site and isolate number	Salinity ‰
**Dead Sea (DS)**	
Water: DSw6, DSw22	370
Sediment: DSs30, DSs34, DSs36, DSs38, DSs40, DSs46,	450
DSs54	

**Estuary of Mandovi (EM)**	
Station 2 water: EM2w_b_107	35
Station 4 water: EM4w_s_118, EM4w_s_120, EM4w_b_125	33
Station 5 water: EM5w_s_130	31
Station 6 sediment: EM6_s_137	05
Station 7 water: EM7w_s_139, EM7w_b_143,	
sediment: EM7s145	10
Station 8 water: EM8_w_s_146, EM8w_s_147, EM8w_s_148,_	16
sediment: EM8s153	10
Station 9 sediment: EM9s156	10

Mangrove water: MRw204, MRw207	32

Salterns water : SCw255	230

### HALOTOLERANCE CURVES

Salt tolerance curves were performed as given by Nazareth et al. (2012). Conidial suspensions of isolates were spot-inoculated on CzA containing solar salt (0–30%). Growth was recorded in terms of colony diameter after 7 d incubation, or after 15 d for those showing delayed growth.

### DETERMINATION OF OBLIGATE REQUIREMENT OF SALT FOR GROWTH ON DIFFERENT MEDIA

Isolates were selected on the basis of their halotolerance curves, and conidial suspensions, 10^3^ spores in 5 μl, were spot inoculated, in triplicate, on Czapek Dox Agar (CzA), Malt Extract Agar (MEA), Potato Dextrose Agar (PDA) and Sabouraud Agar (SA; HI Media), each without and with 10% solar salt (S), to confirm the obligate requirement of salt for growth. Growth was measured in terms of colony diameter after 7 days incubation at 30°C.

### EXAMINATION OF CONIDIAL GERMINATION

Conidial suspensions of selected isolates were spread on to plates of CzA and CzA + 10% solar salt, and incubated in the dark, at 30°C. Three agar plugs were aseptically sampled from each plate of CzA, at 3 h intervals between 12 and 48 h. The agar plug was placed on a slide, stained with lactophenol cotton blue dye and examined microscopically. A total of 50 conidia per agar plug were counted; conidia were considered germinated when the germ-tube length was equal to, or longer than, the diameter of the conidium (Ramirez et al., 2004) and expressed in terms of % germination.

### MORPHOLOGICAL CHANGES IN RESPONSE TO PRESENCE OR ABSENCE OF SALT

Conidial suspensions were spot-inoculated on CzA and on CzA + 10% solar salt, and incubated at 30°C for 15 d. Wet mounts of the isolates prepared in 1:1 lactophenol cotton blue dye (HI Media) were then viewed microscopically for morphological changes in the germination of the conidia, the mycelia and conidiating structures; where growth was not visible on agar media without salt, an agar plug obtained as detailed above, was used for microscopic examination.

## RESULTS

### SALT TOLERANCE CURVES

**FIGURE 1:**
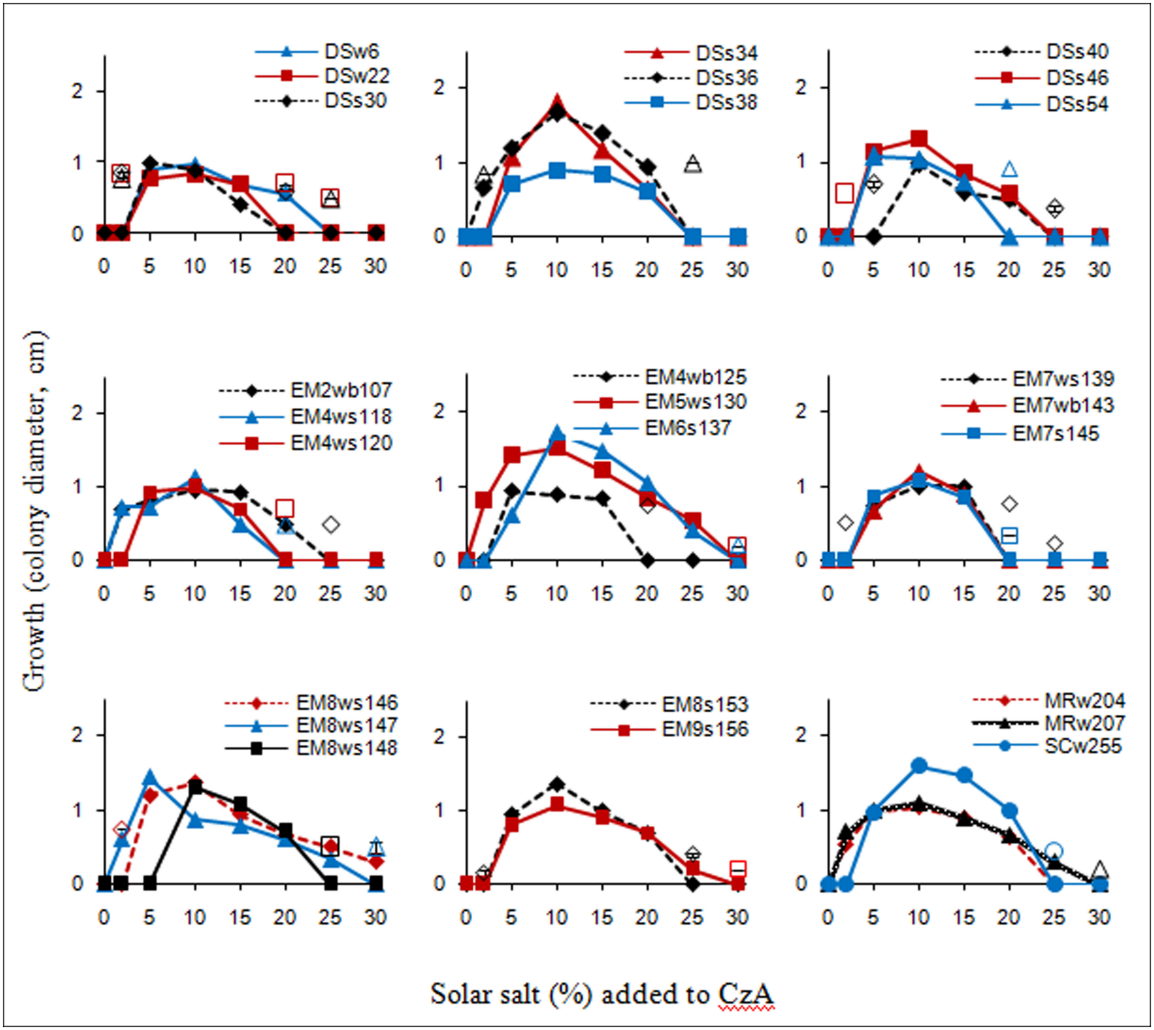
**Salt tolerance curves of the isolates as recorded after 7 d incubation; unconnected open symbols indicate delayed growth at respective salt concentrations, after 15 d incubation**.

The salt tolerance curves of the *A. penicillioides* isolates are shown in **Figure 1**. The results indicate that most of the isolates tested had a minimum salt requirement of 2 or 5% for growth, while a few required 10%, which clearly demonstrated their true halophilic nature. Optimal growth for almost all isolates was obtained at a salt concentration of 10%, with a few growing best at a salt concentration of 5 or 15%, irrespective of the econiche or its hypersaline or polyhaline characteristic from which the isolates were obtained. These isolates were therefore termed as moderate halophiles, in accordance to the definition of Kushner (1978). The isolates were euryhaline in nature, able to adapt to a wide range of salt concentrations, with only one isolate showing a stenohaline nature, having growth over a short range of salt concentrations.

### DETERMINATION OF OBLIGATE REQUIREMENT OF SALT FOR GROWTH ON DIFFERENT MEDIA

One isolate from each of the econiches was selected for the study, on the basis of its greater halophilic nature, having the highest minimal salt concentration requirement for growth, and the highest limit of salt tolerance, all requiring 10% salt concentration for optimal growth: DSs40 which grew readily with 10–20% salt concentrations, with a delayed growth in presence of 5 and 25% salt; EM6s137 which grew with a range of 5–25% salt and delayed growth with 30% salt; MRw207 which grew with salt concentrations of 2–25 or of 30% with a delayed growth.

**FIGURE 2:**
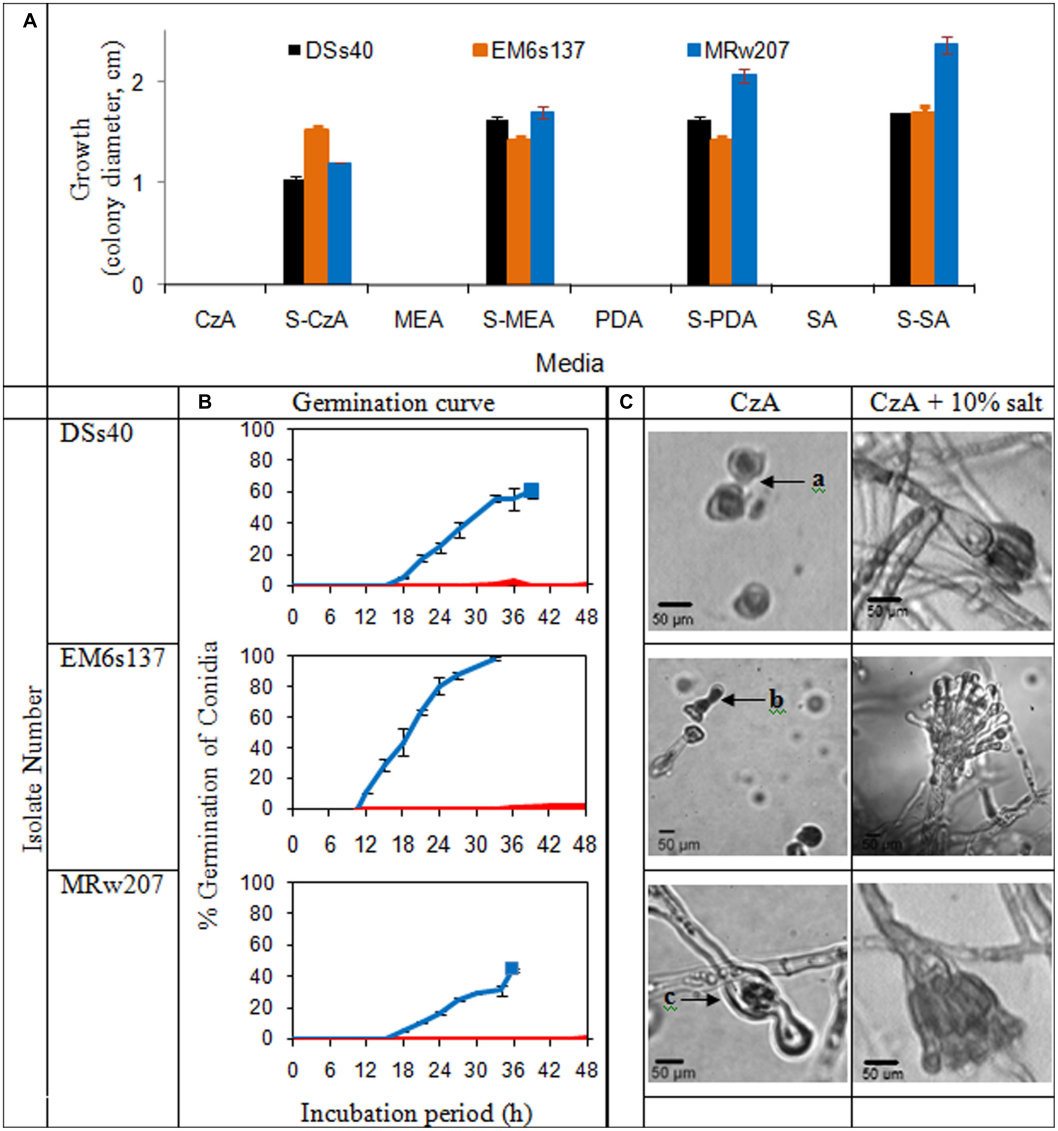
**(A)** Colony characteristics of *Aspergillus penicillioides* isolates on agar media: Czapek Dox (CzA), Malt Extract (MEA), potato dextrose (PDA), Sabouraud (SA), without and with 10% salt (S); **(B)** Curves of conidial germination on CzA amended with salt: 0% (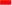) and 10% (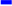). Solid blocks indicate termination of experiment; **(C)** Micromorphology of the isolates after 15 d incubation on CzA and CzA + 10% salt, showing (a): ungerminated swollen and distorted conidia, (b): conidia germinated and distorted, (c): conidia germinated and mycelia distorted with little cytoplasm, and lysis

The growth of the isolates on various nutrient media in presence or absence of added solar salt is given in **Figure 2A**. In absence of added salt, all the isolates tested were unable to grow on chemically defined medium of CzA, as well as the more nutrient-rich media of MEA, PDA and SA. However, with addition of 10% salt, growth was visible on all these media.

### CONIDIAL GERMINATION AND MORPHOLOGICAL CHANGES IN RESPONSE TO SALT

Germination curves and micromorphological examination of the selected isolates are shown in **Figure 2B**. Conidia of DSs40 did not germinate on media without solar salt within the 48 h tested, except for an occasional occurrence. EM6s137 conidia showed 2% germination which was initiated from 36 h; no germination was seen in conidia of MRw207. However, at 10% salt concentration which supported maximal growth, the conidia of all isolates showed germination. DSs40 and MRw207 conidial germination began within 15 h incubation, but while a number of germinated conidia even produced mycelial forms, some conidia had not yet germinated. As the mycelia covered the entire viewing field, making further observations impossible, the study was discontinued. The conidia of EM6s137 showed 100% germination within 33 h.

When incubated on medium without salt, DSs40 conidia remained ungerminated even after 15 days, but the conidia appeared swollen and distorted. Conidia of EM6s137 germinated, but became distorted without further mycelial growth. MRw207 conidia germinated, but formed distorted mycelia with very little cytoplasm, lysis at some parts and with oozing of the cytoplasm. However, at 10% salt concentration that supported optimal growth, micromorphological analysis revealed that not only had conidial germination occurred, but that there was formation of mycelia and vesicles with conidiation, which appeared normal.

## DISCUSSION

The strains of *A. penicillioides* had an obligate requirement of NaCl for growth, as was seen from the halotolerance curves, and were therefore classified as true halophiles. This was supported by the observation that the isolates did not show any growth on synthetic as well as nutrient rich agar media in absence of NaCl, but grew well on the same media when supplemented with 10% solar salt, irrespective of the synthetic or nutrient rich nature of the media, although the latter amended with salt, supported a better growth, possibly providing substrates for synthesis of osmolytes to combat the low a_w_ environment.

The absolute requirement for salt by the isolates was further confirmed by the lack of conidial germination and/or distortion of conidia or the germ tube when incubated in absence of salt, while germination and growth was observed to be normal when grown in presence of salt.

It has therefore been shown that salt and a low a_w_ are essential for the germination of the conidia, as well as germ tube elongation, prior to growth of the culture. The strains of *A. penicillioides* which were obtained from diverse econiches such as the hypersaline athalassohaline Dead Sea and thalassohaline solar salterns, and the polyhaline estuary and mangroves, has thus been shown to be true halophiles.

The basidiomycete *Wallemia ichtyophaga* is another true halophile that requires a minimum of about 9% NaCl for growth and 15–20% NaCl for optimal growth (Plemenitas et al., 2014). However, the strains of *A. penicillioides* are the only asexual filamentous fungi reported thus far.

The osmoadaptation mechanism in true halophiles forms an intrinsic part of its metabolism. A mitogen-activated protein kinase or MAPK pathway, involved in germ tube elongation, branching, and hyphal fusion events between conidial germlings (Pandey et al., 2004), has also been shown to be responsible for transcription of enzymes involved in glycerol synthesis and intracellular glycerol accumulation in response to osmotic stress (Jin et al., 2005). It appears therefore, that in true halophiles, the MAPK pathway is stimulated by conditions of low a_w_, and hence in the absence of such stimulatory conditions, osmoadaptation for germination and/or germ tube elongation does not occur.

True or obligate halophiles can be termed as specialists, with their growth optimum shifted toward extreme values, and have a narrow ecological amplitude (Gostincar et al., 2010). *A. penicillioides* species does not have a sexual life cycle (Tamura et al., 1999) which consequently inhibits gene flow. This will have caused a rapid fix of genetic information in these populations that have managed to adapt to saline habitats (Gostincar et al., 2010).

*A. penicillioides* strains, by means of their absolute requirement for salt, are indigenous to the marine environment (Mackay et al., 1984) and have been shown to exist in diverse econiches**–** from hypersaline to polyhaline systems, from athalassohaline to thalassohaline environments, and at a longitudinal distance of approximately 38.5° apart on the Asian continent. Hence it can be expected to be found globally, in diverse saline econiches.

*A. penicillioides* has been described as xerophilic (Tamura et al., 1999) and osmophilic (Wasser et al., 2003). Its capacity to grow in environments in which the lowering of a_w_ is contributed by sodium chloride ions, as shown above, establishes these isolates as halophilic, as defined by Kushner (1978) and Grant (2004). It is therefore suggested that the species may be polyextremophilic in nature, the low a_w_ being the basic requirement for growth of the species, whether contributed by low moisture, high sugar concentrations, or increased levels of sodium chloride.

## Conflict of Interest Statement

The authors declare that the research was conducted in the absence of any commercial or financial relationships that could be construed as a potential conflict of interest.
